# The development of an automatic speech recognition model using interview data from long-term care for older adults

**DOI:** 10.1093/jamia/ocac241

**Published:** 2022-12-10

**Authors:** Coen Hacking, Hilde Verbeek, Jan P H Hamers, Sil Aarts

**Affiliations:** Department of Health Services Research, CAPHRI Care and Public Health Research Institute, Faculty of Health Medicine and Life Sciences, Maastricht University, Maastricht, The Netherlands; The Living Lab in Ageing & Long-Term Care, Maastricht, The Netherlands; Department of Health Services Research, CAPHRI Care and Public Health Research Institute, Faculty of Health Medicine and Life Sciences, Maastricht University, Maastricht, The Netherlands; The Living Lab in Ageing & Long-Term Care, Maastricht, The Netherlands; Department of Health Services Research, CAPHRI Care and Public Health Research Institute, Faculty of Health Medicine and Life Sciences, Maastricht University, Maastricht, The Netherlands; The Living Lab in Ageing & Long-Term Care, Maastricht, The Netherlands; Department of Health Services Research, CAPHRI Care and Public Health Research Institute, Faculty of Health Medicine and Life Sciences, Maastricht University, Maastricht, The Netherlands; The Living Lab in Ageing & Long-Term Care, Maastricht, The Netherlands

**Keywords:** automatic speech recognition, long-term care, artificial intelligence, older adults, nursing homes

## Abstract

**Objective:**

In long-term care (LTC) for older adults, interviews are used to collect client perspectives that are often recorded and transcribed verbatim, which is a time-consuming, tedious task. Automatic speech recognition (ASR) could provide a solution; however, current ASR systems are not effective for certain demographic groups. This study aims to show how data from specific groups, such as older adults or people with accents, can be used to develop an effective ASR.

**Materials and methods:**

An initial ASR model was developed using the Mozilla Common Voice dataset. Audio and transcript data (34 h) from interviews with residents, family, and care professionals on quality of care were used. Interview data were continuously processed to reduce the word error rate (WER).

**Results:**

Due to background noise and mispronunciations, an initial ASR model had a WER of 48.3% on interview data. After finetuning using interview data, the average WER was reduced to 24.3%. When tested on speech data from the interviews, a median WER of 22.1% was achieved, with residents displaying the highest WER (22.7%). The resulting ASR model was at least 6 times faster than manual transcription.

**Discussion:**

The current method decreased the WER substantially, verifying its efficacy. Moreover, using local transcription of audio can be beneficial to the privacy of participants.

**Conclusions:**

The current study shows that interview data from LTC for older adults can be effectively used to improve an ASR model. While the model output does still contain some errors, researchers reported that it saved much time during transcription.

## INTRODUCTION

In recent years, client perspectives have become increasingly important in healthcare research.^1^ For example, in long-term care (LTC) for older adults, the perspectives of residents, family, and care professionals have become a valuable source of information about the quality of care and quality of life.^2^^,^^3^ To assess these perspectives, often narrative data are collected. Narrative data can be defined as stories that describe the experiences and emotions in someone’s life.^4^ Narrative data can be collected as text (eg, through open text fields in questionnaires) or as audio, acquired by means of recorded interviews.^2^^,^^5^ To be able to objectively analyze the latter type of data, the corresponding audio recordings are transcribed verbatim (ie, written out literally into text).^2^ Currently, in LTC, the transcription of interview recordings is conducted manually by researchers; however, as the number of interviews increases, manual transcription can become very time-consuming and costly. An alternative approach could rely on automatic speech recognition (ASR). ASR is the process of automatically transcribing speech data into written text.^6^

Various studies have shown that English ASR models can achieve a word error rate (WER) below 4%, meaning that of every 100 predicted words, less than 4 words are incorrectly transcribed;^6–12^ however, error rates may be higher when applied in real-world scenarios. Research has shown that for nonnative English speakers, the WERs can be considerably higher, resulting in error rates above 20%.^13^^,^^14^ Moreover, demographic factors such as a person’s age, gender, or accent can influence speech features (eg, pitch, clarity), which increases error rates. For example, older adults are more likely to suffer from speech impairments, possibly leading to audio recordings that are more difficult to recognize by ASR systems.^15^^,^^16^

To improve the accuracy, and thus reduce WERs of current ASR approaches, a more diverse dataset is required, such as data describing a higher diversity in types of voices and accents. More diverse data can lead to an ASR model that is better at recognizing speech irrespective of accent, age, or other demographic factors. To increase the diversity of data, a novel data collection method should be employed. As interview data are abundantly available in healthcare research, data could be collected from interview recordings with corresponding transcripts. For example, such data could include interviews with residents, formal and informal caregivers, and healthcare managers; however, extensive preprocessing is required to be able to use interview data for developing an ASR model with a lower WER. For example, full-length interview recordings are too long for training an ASR model. Thus, audio should be split into segments of an appropriate length before they can be used for the improvement of an ASR model.^7^^,^^8^

Current ASR models still display high WERs for some languages, such as Dutch, or for speech stemming from people with specific accents. Therefore, this study aims to investigate how data from specific demographic resources could be used to develop an ASR model that achieves lower WERs regardless of age, accent, or other speech features. In addition, we aim to investigate the time-effectiveness of the method, compared to the current baseline of manual transcription.

## METHODS

**Figure 1.**
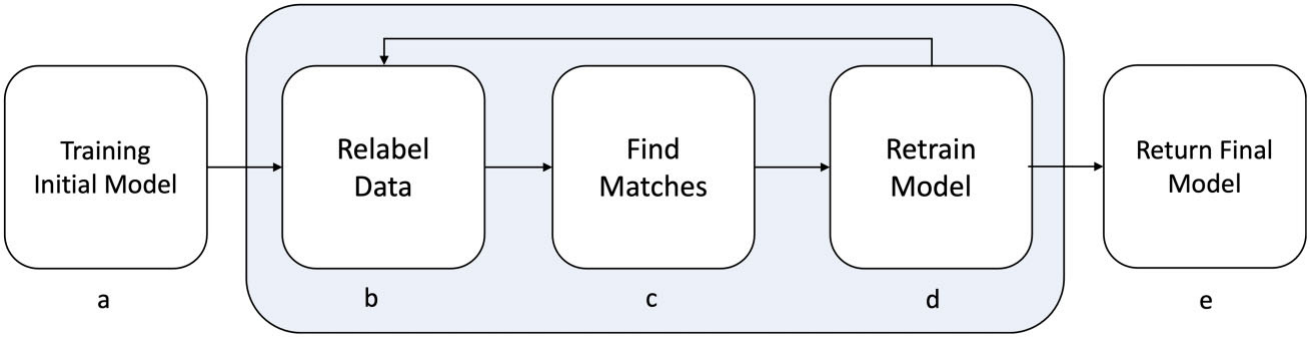
An overview of the iterative automatic speech recognition (ASR) model training process.

To develop an ASR model, speech data were collected from existing datasets, such as the Mozilla Common Voice NL (MCV-NL) corpus, as well as interview data from LTC for older adults. Preprocessing of interview data was required. The steps of the iterative ASR model training process are outlined in Figure 1: (1) an initial model was trained using existing datasets, (2) transcripts were split into segments of an appropriate length and labeled (ie, text was predicted from speech data) using the previously trained model, (3) interview transcripts are used to provide automatic text corrections, (4) the model was further finetuned using additional data, and finally, (5) training of the ASR model was finished if the WER could no longer be decreased; otherwise, steps 2–4 were repeated until the WER remained the same. After steps 1 and 4, the current state of the model was evaluated by comparing the segments transcribed using the ASR model to the ones that were manually transcribed.

### Sample and participants

A total of 232 interviews were conducted at 5 different LTC organizations in the south of the Netherlands over a span of 2 years. These interviews about the quality of care were conducted with residents, family members, and care professionals. Various types of wards were included, including wards for older people with dementia. In total, 50% of interviews were conducted in the regional dialect (ie, Limburgish). The medical ethical committee of Zuyderland (the Netherlands) approved the study protocol (17-N-86). Information about the study was provided to all interviewers, residents, family members, and caregivers by an information letter. All participants provided written informed consent; residents with legal representatives gave informed consent themselves (as well as their legal representatives) before and during the conversations. See Sion et al^2^ for more information.

### Data collection

Data were collected from various sources, including the “Corpus Gesproken Nederlands” (CGN) dataset, the Dutch “Mozilla Common Voice” (MCV) dataset, and the audio recordings and transcripts from interviews with nursing home residents, their family members, and care professionals. The data from interviews were used to construct a dataset for additional finetuning of the ASR model. The distribution of all datasets is shown in Table 1.

**Table 1.** The various data sources used for creating an automatic speech recognition (ASR) model

Dataset	Preprocessing	Number of hours audio	Number of uttered words
CGN: a dataset of general Dutch data, collected from audiobooks, television, radio, and other sources	No	960	1,855,763
MCV-NL (Mozilla Common Voice Dutch) (Version 6.1)^17^: recorded fragments collected through crowd sourcing with a wide variety of different voices	No	61	187,559
Connecting Conversations: audio collected from interviews about quality of care in nursing home with residents, their family members, and care professionals	Yes	34	99,582
Interviews with informal caregivers, formal caregivers, and healthcare managers	Yes	27	83,239
Miscellaneous interviews from various individuals about long-term care for older adults	No	20	62,286

*Note*: For each dataset, a description of its sources, whether preprocessing was required, and how many hours of data are included.

### Model

In the following sections, several components are discussed regarding the preparation of the ASR model. This includes a description of how preprocessing was applied and how the acoustic and language models were constructed. The acoustic and language models are both parts of the deployed ASR model.

#### Preprocessing

The MCV dataset did not require any preprocessing since it entails a dataset for training and includes dedicated train, validation, and test sets.^8^ The CGN dataset includes transcripts with time codes for the start and end of each utterance. These time codes describe which part of the audio corresponds with a certain piece of text. Therefore, the time codes were used to split the audio into smaller audio segments with the corresponding text, suitable for deep learning.

For the interview data, only full-length audio files and full-length transcripts without timestamps exist. This would cause issues when trying to train an ASR model as deep learning requires small segments to learn from. Splitting up the speech data is necessary to be able to fit it into memory and avoid exceeding the maximum size (ie, token limit) the model can process^7^^,^^8^; however, splitting mid-sentence can increase the WER of the ASR model. Therefore, it is important to optimize the time stamps at which the audio is split. To achieve this, several steps were applied, similar to the “Noisy Student” method.^18^ An initial model, $M_{0}$, was trained without the interview data. The interviews were then split into smaller segments, *S* (ie, smaller than 20 s). Then, these segments were transcribed using $M_{0}$, leading to predictions, *P*. These predictions were then aligned with the preexisting manual transcripts using the Smith–Waterman algorithm. The Smith–Waterman algorithm is a local alignment algorithm, which can be used to find text similar to predictions in the manual transcripts. If the similarity was below *p* = 90%, the text segment was discarded. Otherwise, if the text of both was very similar or the same, we assumed that the prediction was correct. Then the manual segments were used as corrections, *P*′. Finally, $M_{0}$ is finetuned using *S* and *P*′. This process is repeated until the WER remains the same between iterations (ie, no additional improvement is possible).

For ASR, it is important that audio segments used for training are short enough to for the model, which may only support speech segments of a limited length.^8^^,^^9^ Therefore, some of the audio files required preprocessing to facilitate the development of an ASR model, in the form of splitting and text alignment. Segments that are too short do not contain the information necessary to develop a speech recognition model, while segments that are too long may become more difficult to align with the corresponding part in the audio and may require too many computational resources. It is therefore essential that interview recordings are split into segments; however, splitting the audio at static intervals may result into splitting mid-word or mid-sentence, which could increase the WER.^19^^,^^20^ Therefore, the audio of the interviews was split between occurring silences. This process was conducted iteratively, initially splitting on a minimum silence length of 250 ms and repeating this each time with half the minimum silence length until all segments are of the correct length.

#### Acoustic model

An acoustic model predicts a token for every 10 ms of the audio (with a small amount of overlap) and the probabilities for every possible token in the vocabulary (eg, all of the characters in the alphabet). A token is an index representation of a character (eg, 0 = “a,” 1 = “b”).^8^ The acoustic model returns a probability for each possible token, and the token with the highest probability is selected (eg, given *P*(0) = 0.9 and *P*(1) = 0.8, then token 0 is selected). This results in a list of tokens, which are then decoded into a piece of text. For example, a 0 is turned back into the letter “a.”

In this study, the HuBERT methodology was used for the acoustic model.^8^ This methodology shows that a model can be pretrained using a clustering approach on a large audio dataset without the need for corresponding transcripts. This pretraining allows the model to learn what speech features are important to make correct text predictions.^8^ An English version of the model was used as a starting point for developing a Dutch model.

#### Language model

When transcribing text, it is not always possible to understand every single word, neither for speech recognition software nor for humans. It is often necessary to rely on language information, such as knowledge about what sentences are grammatically correct, to understand imperfect speech data. For ASR, this is achieved by using a language model. Language models are models that can encode the probability of (sequences of) words.^21^^,^^22^ For example, a sentence such as “my dog likes the park” is more probable to occur than “the park likes my dog.” As another example, given a sentence such as “my dog likes the [blank],” a language model can predict which word best fits the blank. By leveraging the language information, it becomes possible to understand sentences, even when not all words could be understood from the speech data. Thus, a language model can be used to correct errors using language information (eg, words that are similar or ambiguous based on their pronunciation).^8^ For example, the words “then” and “than” are pronounced exactly the same but have different spellings and functions in a written sentence. In this study, RobBERT, a preexisting language model, was used.^22^

### Analyses

To establish whether the proposed method is effective in finetuning an ASR model for the purpose of transcribing speech data in LTC for older adults, the method was evaluated in 3 different ways. First, the accuracy of the model was evaluated by measuring if there is a significant decrease in WER. The WER was evaluated at the initial stage and at every successive iteration. The WER was obtained by evaluating the MCV-NL validation set. If the measured WER values decrease over time, it is an indication that the method may be effective. To ensure that the change in WER was only due to an improvement in the accuracy of the model, the validation set remained the same during all iterations of the process. To further test the effectiveness, interview transcript predictions of the final ASR model were compared to manual transcripts in terms of overlapping words. Second, the time-effectiveness of the resulting model was analyzed to establish if using the model, in practice, could reduce the time required to transcribe. Lastly, an error analysis related to insertions (ie, any character that was wrongfully added to the transcript), deletions (ie, a character is missing from the transcript), and substitutions (ie, a character was wrongfully exchanged for another).^23^ The ratio of individual error types compared to the total amount of errors was calculated.

## RESULTS

### Accuracy

At first, a model was trained using the CGN dataset. This model achieved a WER of 4.6% on its own validation set. A validation set is used to validate the accuracy of a model. However, when validated on speech data from LTC (ie, the interviews), this model only achieved a WER of 48.3%. A second model, trained only on the MCV-NL dataset, achieved a WER of 23.6% on its own validation set. When applied to speech data from LTC, this model achieved a WER of 37.6%. This indicates that the first model might have been trained too strongly on Dutch with a typical accent. The second model went through several iterations of the proposed method (see Table 2). Every iteration improves the WER of the model and increases the number of correctly recognized segments.

**Table 2.** The number of correctly recognized interview samples included in each iteration and the resulting word error rate (WER) of the validation set

Iteration	# samples	WER (%)
0 (initial)	0	22.9
1	1137	21.6
2	2253	20.3
3	3429	19.4
4 (final)	4567	17.3

**Figure 2.**
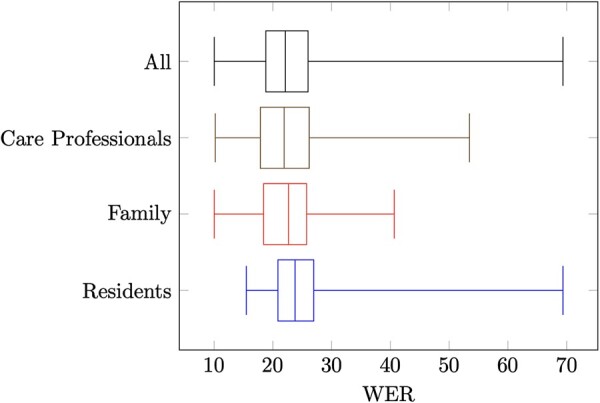
Results of the automatic speech recognition (ASR) model when applied to interview data. For each group (residents, family, and care professionals), the word error rates (WERs) are plotted (*x*-axis).

For all segments in the interview data, the WER was calculated. The distribution of these scores has been visualized as boxplots for each of the different groups (ie, residents, family, and care professionals) in Figure 2. These results show that audio from residents shows the highest WERs, which means that the speech of this group is the most difficult to recognize, with a median of 23.8% correctly aligned text. Family members show a better WER with a median of 22.6%, and care professionals have the best ratios with a median of 21.9%. All groups are shown to have some outliers (ie, individual cases that are far away from other data points) on the high and low end; none of the interviews surpass a WER of 10.0%. The WER on data from the interviews went from 48.3% after training on CGN to 37.6% after iteration 0 (ie, where only data from MCV-NL were used) and then to 23.7% after iteration 4 (ie, where data from interviews have been used 4 times).

### Time-effectiveness

When manually transcribing speech data (ie, interviews), the speed of transcribing is limited to various factors, such as the playback speed of the audio. At 1× speed (ie, not faster than recorded), it would be possible to transcribe at most 60 minutes (of audio) per hour (of transcribing). It could be argued that this is an upper limit, as manual transcription may require listening to certain parts of the audio multiple times. With this same logic in mind, when playing the audio back at 10× speed, at most 600-minute audio per hour could be transcribed manually. No person could type this fast, and this is still significantly slower compared to transcription conducted through ASR. In our experiments, transcription speeds were assessed using an Nvidia RTX 2060. Using this hardware configuration, an average transcription speed of 3618-minute audio per hour was reached; however, as the validated output of this transcription had a WER of 22.6%, it should be considered that manual transcription is still required to achieve a 100% correct transcript. For example, in cases where there is a lot of noise in an audio segment or where people are talking at the same time, manual transcription would still be needed.

Various researchers from the Living Lab in Ageing and Long-Term Care in Limburg, The Netherlands were able to use the method by means of a web application. The researchers reported that the application was of added value; the tediousness of verbatim transcription is reduced to making small corrections to the automatically generated transcripts. Some researchers noticed that certain accents could still result in transcription errors, which required more manual correction. “not everything is transcribing correctly, but in most cases I can correct the misspellings without listing to the audio.” Interestingly, researchers sometimes reported that even they could not always transcribe the audio, the primary reason being background noise and a lack of clarity in the voice of an interview participant.

### Error analysis

The error analysis (Table 3) indicates that insertions are responsible for the majority of the errors; in the majority of cases, the ASR model inserted an additional character compared to the manual transcript. In contrast, a relatively low ratio of substitutions is observed (ie, a character is wrongfully replaced by another character). The ratio of deletions may indicate that words are not always recognized.

**Table 3.** The distribution of error types of the automatic speech recognition (ASR) model from manual to predication^a^

	Total	Residents	Family	Care professionals
Insertions (%)	70.97	73.87	73.11	76.19
Substitutions (%)	8.91	7.29	8.25	6.66
Deletions (%)	20.12	18.84	18.64	17.16

a The ratio of insertions indicates where the ASR model wrongfully added a character. The number of substitutions indicates where a character was wrongfully recognized. The ratio of deletions indicates where the ASR model wrongfully did not add a character to the output.

## DISCUSSION

The present study investigated how data from specific demographic groups could be used to develop an ASR model applicable in daily care practice and academia. This method decreases WERs regardless of specific demographic characteristics, such as accents, age or speech problems. Results show that interview data stemming from an LTC setting can be transformed into a useful resource for improving the recognition performance of an existing ASR model.

The current results indicate that by iteratively processing and adding more interview data, the WER of the ASR model was reduced; every successive iteration of the method was able to recognize more samples (ie, pairs of audio and text segments) from the interview data, eventually leading to a WER of 17.3%. These results imply that the process of iterative refinement is indeed effective for deploying interview data to decrease the WER of existing ASR models. Besides being tested on the MCV-NL dataset, the ASR model was tested on the interview data stemming from residents, family members, and care professionals. This resulted in slightly higher WERs. This may be due to various factors such as background noise, which is already known to increase WERs.^6–8^

In addition, although differences appear to be small between the 3 groups (ie, residents, family members, and care professionals), the highest WERs were displayed by residents. This could be related to the age of the persons in this group; since residents are the group with the highest average age, they are more likely to suffer from speech problems.^24^

While part of the error of a model could be explained by the limitations of the ASR model itself, errors in manual interview transcripts could also have resulted in a higher WER (comparing an ASR model output to an incorrect transcript will still be perceived as an error of the ASR model). While transcripts ideally are a completely accurate presentation of the verbatim utterances of interview recordings, transcripts are not perfect text replications of the audio at hand. Manually transcribed interviews may exclude mispronounced words, thinking noises (eg, uh, uhm), privacy-sensitive information (eg, names of the interview participant), and words and utterances that could possibly not be understood by the person transcribing the interview.

While there are commercially available systems that appear to achieve lower WERs than the WER conducted in the current study, these services often process data in the cloud (ie, off-site). Since interviews conducted in a nursing home setting involve some of the most vulnerable people in our society, care and research organizations may require its processing to be conducted within the organization, rather than to hand it to a commercial party (eg, Google or Microsoft). Hence, developing ASR models for use within an organization may be necessary to guard the privacy of interview participants.

An analysis of the time-effectiveness showed that the ASR model transcribed at least 6 times faster than manual transcription. Consequently, a large part of the manual work that is required for transcription can be reduced using the method introduced in the current article. Feedback from researchers at the Living Lab in Ageing and Long-Term Care mentioned that while transcription results were perceived useful, factors like background noise being present resulted in a higher WER, and thus, more errors in transcription which had to be corrected manually. However, many errors were small (ie, related to the insertion of a letter) and transcripts could be understood “as is.”

The error analysis shows that few substations occur, which may indicate that words that are recognized are generally spelled correctly. The number of insertions may correspond with nonlexical words, but also words that were omitted from the manual transcript and false positives from background noise. Cases have been observed where the ASR model recognizes words that were missing from the manual transcript. For example, the ASR model transcribed the following text “fthe last question is there anything you want to tell …,” whereas the word question was omitted in the transcript “the last—is there something you want to tell ….” In this case, the only error of the ASR model is the first “f” in the sentence, while other characters are wrongfully regarded as errors. The number of deletions corresponds with the observed behavior of the ASR model where the beginnings and ends of segments are not correctly recognized. Although in all cases of errors, noise is observed to be a large factor. By using more advanced recording hardware: by using microphones that are less sensitive to background noise, the WER of the ASR model on those audio recordings may be reduced. In addition, research has shown that many languages share similar speech features.^25^ Consequently, the current method of sampling may not only be applicable to the current ASR model but may also be applied to finetune ASR models aimed at other languages. When comparing the approach in the current study to the results of state-of-the-art methods for English ASR, the WER above 20% on interview data may seem high; however, the difficulty of the speech in this dataset may contribute to a higher WER. The robustness (ie, how well an ASR model performs across different datasets) may still be low even if the WER for 1 dataset is also low. For example, the English HuBERT model has a reported 1.9% WER on the LibreSpeech-clean dataset; however, it has a 58.5% WER on the CHiME6 dataset.^25^

Although the method of iterative improvement of the ASR model was shown to be effective and the resulting ASR model was viewed as a useful tool when transcribing interview recordings, the method also has its limitations. First, the Smith–Waterman alignment is used to reduce the WER of text segments from the ASR model. While the Smith–Waterman approach of alignment does ensure a certain quality of data, text segments may still contain errors. This could potentially be improved by giving more context (ie, words surrounding the text segment) to the ASR model to learn from.^19^^,^^26^ A second limitation regards the size of the model. Research implies that to be able to learn more nuanced speech features and reduce the WER of an ASR model, the size of that model and dataset should be increased^27^^,^^28^; however, training and running a larger ASR model than the one discussed here would also require more computational resources, leading to higher costs for the use of the ASR model in daily practice.^29^

In the Dutch language, the pronunciation of words may change based on used accent marks, such as “*é*” and “*ö*.” In the present study, this was not taken into account, as the ASR model was finetuned from an existing English model. The model used the characters A–Z, as well as dashes and apostrophes. Future research efforts should focus on using methods that employ characters with accent marks. This could further reduce the WER of the ASR model, as in the current study accent marks are removed, which also reduces the information that the ASR model receives.

In addition, research could focus on verifying if the text produced by the ASR is interpretable for qualitative analyses, to further validate the practical usage of the ASR model presented in this study. Currently, when interviews are conducted in LTC for older adults, these are transcribed and analyzed by hand. In these analyses, themes relevant to a research question are identified within the interviews.^30^ When applying the WER metric, sentences which may have a high WER may still be understandable to human readers, and the qualitative analyses (ie, the identification of themes) can still be conducted without producing erroneous results. For example, if each word in a sentence would be slightly misspelled, it could still be interpreted without problems. Thus, the WER does not have to be zero for a successful qualitative analysis.

The current study developed and assessed an ASR model for usage on conversational data such as interviews regarding the quality of care and quality of life. Research should also focus on finetuning an ASR model for clinical usage. Such a model would be used for speech data that contains clinical or medical terms (eg, speech data in electronic health records).

## CONCLUSION

This study shows that full-length interview data (consisting of audio and corresponding transcripts) can be effectively used to improve ASR. This was validated using data stemming from interviews with residents, family members, and care professionals from LTC for older adults. Relatively few real-world samples from interviews can contribute to reduction of the WER for demographic groups with less common speech features, such as older adults. While ASR can certainly reduce the amount of time and (inherently) cost that are required in the manual transcription of audio, the results of the developed ASR system cannot yet compete with manual transcription; however, the tediousness of verbatim transcription is reduced to making (often small spelling) corrections to automatically generated transcripts.

## Data Availability

The code and models discussed in the article will be made available at https://github.com/coen22/Speech-Recognition. Our interview data will not be publicly available due to the privacy of our participants. Upon request, our interview data may be provided with restrictions. Data are available from the Living Lab in Ageing and Long-Term Care (contact via Sil Aarts; s.aarts@maastrichtuniversity.nl) for researchers who meet the criteria for access to confidential data.
